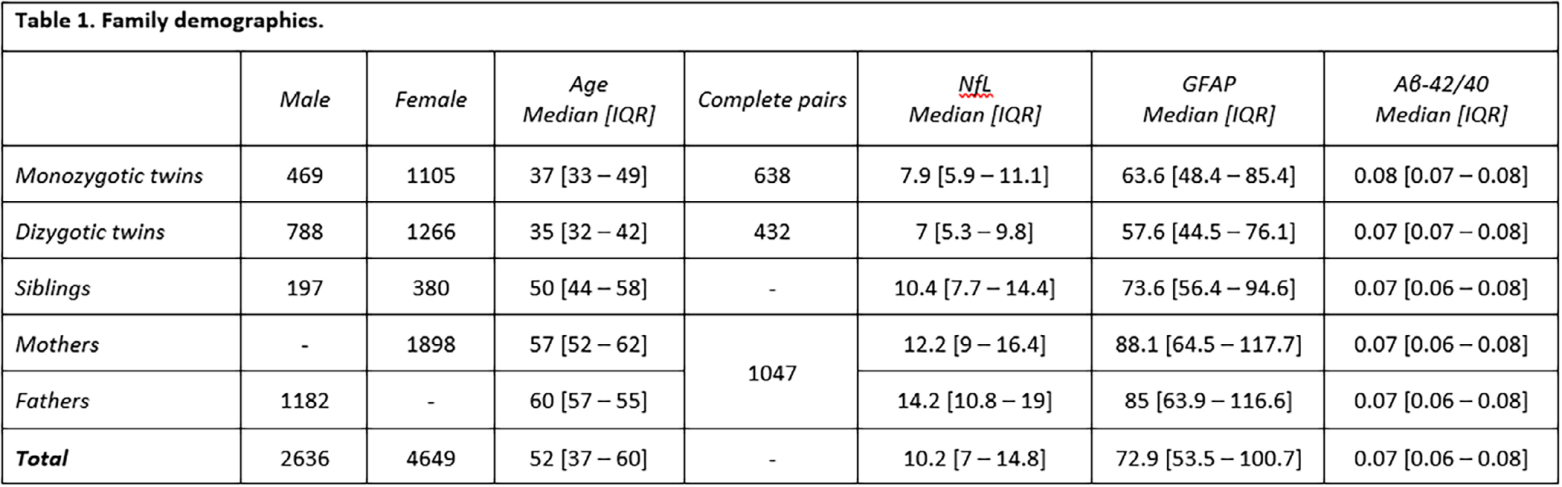# Heritability of plasma biomarkers for Alzheimer’s Disease: A Nuclear Twin Family Design

**DOI:** 10.1002/alz.090338

**Published:** 2025-01-09

**Authors:** Rebecca Z. Rousset, Anouk den Braber, David H Wilson, Charlotte Teunissen, Eco J.C. de Geus

**Affiliations:** ^1^ Neurochemistry Laboratory, Amsterdam UMC location VUmc, Amsterdam Netherlands; ^2^ Amsterdam UMC, Amsterdam Netherlands; ^3^ Department of Biological Psychology, Vrije Universiteit Amsterdam, Amsterdam Netherlands; ^4^ Quanterix, billerica, MA USA

## Abstract

**Background:**

Plasma biomarkers may be a non‐invasive and cost‐effective tool for diagnosing Alzheimer’s Disease. Promising markers include amyloid‐β‐42/40 ratio (Aβ‐42/40), glial fibrillary acidic protein (GFAP), and neurofilament‐light (NfL). Not much is known about what genetic and environmental factors influence and potentially confound these biomarker levels. We aimed to determine the proportion of genetic (heritability) and environmental contributions on Aβ‐42/40, NfL, and GFAP plasma levels.

**Method:**

Male and female twins, their parents, and up to two siblings were included (n=6516, Table 1). Plasma samples were analyzed on the Simoa HDx analyzer using the Neurology 4‐Plex E advantage kit (Quanterix). Biomarker levels were adjusted for age and sex. A twin‐only 5‐zygosities model was used to examine sex differences in genetic and environmental effects. A nuclear twin family design (NTFD) was used to estimate the proportional contributions of genetic, shared and unique environment. Shared environmental factors were broken down into environment shared by siblings, by twins only, or by parents and offspring.

**Result:**

No sex differences in contributions to the variance in biomarker levels were found. The NTFD showed that 16% of the variance in plasma Aβ‐42/40 was explained by genetic effects and 84% by unshared environment. For NfL, 30% of variance in plasma levels was explained by genetic effects, 27% by sibling‐shared environment, and 43% by unshared environment. For GFAP, 45% of the variance in plasma levels was explained by genetic effects, 17% by sibling‐shared environment, and 38% by unshared environment.

**Conclusion:**

The heritability of plasma Aβ‐42/40 was low, while the heritability of NfL and GFAP levels was moderate. The low heritability of Aβ‐42/40 is a mismatch with the high heritability of Alzheimer’s Disease (60‐80%), suggesting this plasma ratio might be susceptible to environmental confounding. The importance of sibling‐shared environment could be a reflection of a generational effect. If members of a generation (siblings) are collectively more exposed to a factor which influences their plasma levels, that would make them resemble each other more than they resemble the previous generation (parents). This findings are not definitive proof of confounding, but are a first step in identifying possible confounders.